# Does aortic pulse wave velocity have any prognostic significance in advanced heart failure patients?

**DOI:** 10.15171/jcvtr.2017.05

**Published:** 2017-03-06

**Authors:** Abolfazl Dohaei, Sepideh Taghavi, Ahmad Amin, Shahin Rahimi, Nasim Naderi

**Affiliations:** Rajaie Cardiovascular, Medical and Research Center, Iran University of Medical Sciences, Tehran, Iran

**Keywords:** Arterial Stiffness, Pulse Wave Velocity, Heart Failure, Hemodynamics

## Abstract

***Introduction:*** Noninvasive measurement of arterial stiffness by pulse-wave velocity (PWV) has prognostic value in different sub groups of cardiovascular disorders. We aimed to measure the PWV in advanced heart failure (HF) patients with reduced left ventricular ejection fraction (LVEF) and investigate whether it has any prognostic significance in this group of patients.

***Methods:*** Between 2013 to 2015 patients with a diagnosis of advanced HF (LVEF ≤ 30%) scheduled for right heart catheterization (RHC) were included in our study. PWV was measured before RHC in each patient using vascular explorer device (Enverdis GmbH) in catheterization laboratory. The patients were subsequently followed for 6 months and their hospitalization or death (composite of all-cause death/hospitalization) were recorded.

***Results:*** A total of 50 patients (38 men) were enrolled. The mean (SD) of age was 45 (16) years. The mean PWV was 6.8 m/s. There was no statistically significant correlation between the PWV and the clinical, echocardiographic and RHC data. The PWV was not different in patients with or without composite of all-cause death/hospitalization (7.3 versus 6.3, *P* > 0.05). In this study cardiac output (CO) (beta = -0.53, *P* = 0.02, odds ratio = 0.6, 95% CI = 0.4-0.9), pulse pressure (PP) (beta = -0056, *P* = 0.03, odds ratio=0.95, 95% CI = 0.89-0.99) and age (beta = -0.045, *P* = 0.05, odds ratio = 0.96, 95% CI = 0.9-1.001) were independent predictors of composite of all-cause death/ hospitalization.

***Conclusion:*** In patients with advanced systolic HF, PWV may not be a good prognostic factor and does not have any added value over previous well known prognostic factors.

## Introduction

It has been shown that vascular function is impaired in patients with heart failure (HF). Arterial stiffness or reduced aortic distensibility plays an important role in exercise intolerance of this group of patients.^1-5^ Aortic distensibility can be reliably estimated by aortic pulse-wave velocity (PWV). PWV is the gold standard method for measuring arterial stiffness.^6,7^

Several studies have shown that the PWV is a predictor of cardiovascular and all cause of mortality in a variety of cardiovascular disorders.^8-12^ Some of these studies show that PWV is associated with peak VO2, level of pro-brain natriuretic peptide (BNP) and mortality in patients with HF including those with systolic HF.^3,13-16^

In this study, we sought to measure the PWV value in advanced HF patients with reduced left ventricular ejection fraction (LVEF) who were candidate for right heart catheterization (RHC) and investigate whether PWV had any association with prognostic factors in advanced HF patients.

## Materials and Methods

### Study population

Between 2013 and 2015, among patients referred for RHC, 50 patients were consecutively enrolled according to following inclusion criteria: presence of advanced systolic HF with LVEF ≤ 30% with ischemic or non-ischemic etiology. The indications for RHC was pre-heart transplant evaluation and/or hemodynamic study in advanced HF patients.

Advanced HF was defined according to American college of cardiology/American heart association (ACC/AHA) and European guidelines as; a stage D HF patient who is highly symptomatic most of the times with truly refractory HF symptoms (New York Heart Association [NYHA] class IIIb, IV) despite guidelines directed medical therapies, frequent outpatient visits (≥ 2 visit per months) and recurrent hospitalization (≥ 2 hospitalization or emergent room visits for HF in the past year or ≥ 1 hospitalization in the past 6 months) for decompensation and fluid overload, arrhythmias and complications of HF (worsening renal function, electrolyte imbalance, medication side effects, pulmonary emboli).^17^

Exclusion criteria were as follows: Presence of any arrhythmias including atrial fibrillation (AF) and frequent premature ventricular contractions, acute coronary syndrome, significant valvular heart disease (VHD), congenital heart disease, chronic renal failure, hypo or hyperthyroidism, chronic obstructive pulmonary disease (COPD), diabetes mellitus (DM), and the history of nitrate consumption during the previous week . Patients with other indications of heart transplantation were also excluded. All patients were subsequently followed for 6 months and their re-hospitalization due to cardiovascular events or death was registered.

### Data acquisition and laboratory measure­ments

A comprehensive medical and drug history was taken and thorough physical examination and transthoracic echocardiography was performed by an expert cardiologist. The NYHA function class of each patient was determined by evaluating each patient at rest and different activities including dressing, walking, and climbing the stairs. The scores given to each patient ranged from I (no symptoms) to IV (symptoms at rest).^18^

### Echocardiography

A complete 2-dimensional (2D) color Doppler echocardiogram was performed in each subject in accordance to the American Society of Echocardiography Recommendations^19^ using a commercial GE Vivid 7 with a three-MS variable frequency harmonic phased array transducer just before performing RHC.

LVEF was measured by Simpson’s method. The early diastolic mitral flow velocity (E), early diastolic annular velocity (E’) and E/E’ ratio were measured using standard Doppler imaging. To assess right heart function, the right ventricular tissue Doppler peak myocardial velocity (RVsm), tricuspid annular plane systolic excursion (TAPSE) and tricuspid regurgitation gradient (TRG) were measured.

### Measurement of pulse-wave velocity

PWV was measured just before RHC using Enverdis vascular explorer device (Enverdis GmbH) by an expert cardiologist who was blinded to the patient characteristics. The room was quiet with stable temperature and each patient remained in a resting supine position at least for 10 minutes before the measurement.

### Right heart catheterization

The RHC was performed in catheterization laboratory with 7F balloon tipped pulmonary artery catheter. All measurements were acquired with the patients in supine position and breathing room air at end expiration. The following variables were measured in each patient: mean pulmonary capillary wedge pressure (PCWP), systolic and diastolic pulmonary artery pressure (PAP), mean PAP, right ventricular systolic pressure (RVSP), right ventricular end diastolic pressure (RVEDP) and mean right atrial pressure. The cardiac output (CO) was calculated by estimated Fick method and cardiac index (CI) was calculated by dividing the CO to body surface area (BSA). Stroke volume (SV) was obtained by dividing the CO by heart rate. Brachial blood pressure was measured via a mercury sphygmomanometer in a supine position and after 10 minutes of rest. The pulse pressure (PP) was calculated by subtracting systolic and diastolic blood pressure. The aortic compliance was calculated by dividing the SV to PP.

### Statistical analysis

IBM SPSS statistics 19 for windows (IBM Corp, Armonk, NY, US) was used for all statistical analyses. At first all data were assessed for normal distribution using Kolmogorov-Smirnov test. Quantitative variables were expressed as mean (standard deviation, SD) and qualitative data were expressed as number (percentage, %). To compare different variables student *t* test, analysis of variance (ANOVA), Mann-Whitney U test and chi-square test were used as appropriate. Pearson correlation coefficient (r) or Spearman rank correlation coefficient (rho) was also used to show linear correlations between PWV and hemodynamic measures. Binary logistic regression was used for multivariate analysis. Values of *P *< 0.05 was considered significant.

## Results

A total of 50 eligible patients (12 women and 38 men) were enrolled in our study. The mean (SD) of age was 45 (16) years, between 16-72 years. Non ischemic dilated cardiomyopathy was comprised 28 (56%) of the patients and the rest had ischemic cardiomyopathy.

The most common etiology for non-ischemic dilated cardiomyopathy was idiopathic dilated cardiomyopathy (26 patients) and the etiology in 2 patients was peripartum cardiomyopathy. The mean (SD) of LVEF was 21.8 (8.9).

**Table 1:** Demographic and clinical and echocardiographic findings of study population (n = 50)

**Characteristics**	**Value**
Age, year, mean (SD)	45 (16)
Sex, number (%)	
Female	12 (24)
Male	38 (76)
BSA, Kg/m^2^, mean (SD)	1.8 (0.2)
Diagnosis, number (%)	
Non-Ischemic CMP	28 (56)
Ischemic CMP	22 (44)
NYHA function class, number (%)	
II-III	-
III	14 (28)
III-IV	30 (60)
IV	6 (12)
Drugs and device history, number (%)	
ACE/ARB	50 (100)
Beta blocker	48 (96)
Diuretics	50 (100)
Spironolactone	36 (72)
Digoxin	3 (6)
ICD/CRT	8 (15)
LVEF, median (IQR)	20 (15-30)
RVSm, median (IQR)	8 (6-10)
TAPSE, median (IQR)	11 (10-14)
TRG, median (IQR)	25 (20-35)
E/E’	20 (7)
Pulse wave velocity, median (IQR)	6.8 (3.3)

Abbreviations: ACEI; angiotensin converting enzyme inhibitor, ARB; angiotensin receptor blocker, BSA; body surface area, CMP; cardiomyopathy, LVEF; left ventricular ejection fraction, RV; right ventricle, SD; standard deviation TAPSE; tricuspid annular plane systolic excursion, TRG; tricuspid regurgitation gradient.

**Table 2:** Catheterization findings of study population (n = 50)

**RHC variables**	**Value**
Mean RA pressure, mm Hg, Median (IQR)	12 (8-18)
RV systolic pressure, mm Hg, Median (IQR)	40 (30-50)
RV diastolic pressure, mm Hg, Median (IQR)	7.5 (5-15)
PA systolic pressure, mm Hg, Mean (SD)	45 (15)
PA diastolic pressure, mm Hg, Mean (SD)	24 (11)
PA mean pressure, mm Hg, Mean (SD)	31 (15)
PCWP, mm Hg, median (IQR)	21 (15-32)
Systolic BP, mm Hg, Mean (SD)	108 (15)
Diastolic BP, mm Hg, median (IQR)	72.5 (60-80)
MAP, mm Hg, median (IQR)	87 (73-93)
Pulse pressure, mm Hg, Median (IQR)	38.5 (30-43.5)
Heart rate, bpm, Median (IQR)	93 (80-100)
CO, L/min Mean (SD)	3.9 (0.98)
CI, L/min/BSA, Mean (SD)	2.3 (0.7)
SV, mL, median (IQR)	42 (35-53)
SVR, Wood unit, Median (IQR)	17.9 (14.5-22.1)
PVR, Wood unit, Median (IQR)	1.5 (1.1-3.1)
Compliance.mL/mm Hg, Mean (SD)	1.3 (0.5)

Abbreviations: bpm; beat per minutes; BP; blood pressure; CI, cardiac index; CO, cardiac output; SD, standard deviation; MAP, mean arterial pressure; PA, pulmonary artery; PCWP, pulmonary capillary wedge pressure; PVR, pulmonary vascular resistance; RA, right atrial; RV, right ventricular; SD, standard deviation; SVR, systemic vascular resistance.

### Evaluation of arterial stiffness

In this study, PWV was used for assessment of aortic stiffness. The mean (SD) of PWV was 6.8 (3.3) m/s.

Table 3 shows mean PWV in different subgroups of study. As shown in Table 3, there was no significant difference between different subgroups in terms of PWV. Although the mean PWV was higher among patients with ischemic cardiomyopathy and those older than 50 years old, this difference was not statistically significant.

**Table 3:** Pulse wave velocity values in different subgroups of study, n = 50

**Subgroups**	**PWV, mean (SD)**	**P value**
Diagnosis		0.5
Non-ischemic CMP	6.4 (3)	
Ischemic CMP	7.1 (3.6)	
NYHA class		0.7
III	7.2 (3.1)	
III-IV	6 (3.3)	
IV	6 (3.2)	
Age group		0.5
<50 years	6.4 (2.9)	
≥50 years	7.2 (3.4)	
Combined event		0.2
Yes	7.3 (3.1)	
No	6.3 (3)	

Abbreviations: CMP, cardiomyopathy; NYHA, New York heart association; PWV, pulse wave velocity; SD, standard deviation.

In this study there was also no significant relationship between the PWV and echocardiographic and/or hemodynamic measures (Pearson correlation coefficient or Spearman rank correlation coefficient was between 0.001-0.1 for each analysis with non-significant *P* values)

### Findings of the Patients’ Follow-up

All study population was followed for 6 months after enrolment in the study. No patient was lost during follow up. The composite of all-cause death/hospitalization (occurrence of death or cardiovascular re hospitalization) were observed in 29 (58%) patients.

Seven patients died and 22 patients were admitted due to decompensated HF.

Table 4 shows predictive factors of the composite of all-cause death/hospitalization in bivariate analysis. As shown in Table 4, higher NYHA class, lower systolic blood pressure, SV, CO and index as well as higher right atrial pressure, mean PAP and PCWP were predictors of the composite of all cause death/hospitalization in our study population. The composite of all-cause death/hospitalization were more common among younger patients, because most of them were candidate for heart transplantation and had more critical conditions.

**Table 4:** Predictors of composite of all-cause death/hospitalization in study population, n=50

**Variables**	**Composite of all-cause death/hospitalization**		***P*** ** value**
	**No**	**Yes**	
Age, year, mean (SD)	41 (13.4)	51.7 (14.6)	0.004
Sex (F/M), number	9/21	8/23	0.4
NYHA class			0.001
III	11	3	
III-IV	10	20	
IV	0	6	
LVEF, %, mean (SD)	29.3 (9.3)	19.6 (8.9)	0.001
SBP, mm Hg, mean (SD)	131 (26)	110 (170	0.001
DBP, mm Hg, median (IQR)	80 (70-80)	65 (60-80)	0.2
Pulse pressure, mm Hg, median (IQR)	40 (30-47)	35 (30-40)	0.1
Compliance, mL/mm Hg, mean (SD)	1.2 (0.5)	1.1 (0.5)	0.6
Heart rate, bpm, median (IQR)	92 (76-103)	94 (82-100)	0.2
RA pressure, mm Hg, median (IQR)	10 (6-15)	14 (10-19)	0.03
Mean PAP, mm Hg, mean (SD)	26.8 (15)	34.7 (13.6)	0.03
PCWP, mm Hg, median (IQR)	15 (12-26)	25 (18-34)	0.02
CO, L/min, mm Hg, mean (SD)	5.5 (2.2)	3.9 (1.2)	0.001
CI, L/min/BSA, mean (SD)	3 (1.2)	2.1 (0.6)	0.001
SV, ml, Mean (SD)	66.5 (31)	43.4 (14)	0.001
PVR, Wood unit, median (IQR)	1.5 (1.1-2.8)	1.6 (1.1-3.4)	0.2
SVR, Wood unit, median (IQR)	18.7 (15.2-22.4)	16 (13.4-22)	0.08
RVSm, m/s, mean (SD)	9.2 (2.1)	7.3 (1.7)	0.001
TAPSE, mm, mean (SD)	13.5 (3.6)	11.1 (2.7)	0.004
PWV, m/s, mean (SD)	6.3 (3)	7.3 (3.1)	0.2

Abbreviations: CO; cardiac output, CI; cardiac index, DBP; diastolic blood pressure, LVEF; left ventricular ejection fraction, PA; pulmonary artery, PCWP; pulmonary capillary wedge pressure, PVR; pulmonary vascular resistance, PWV; pulse wave velocity, RA; right atrial, RV; right ventricular, SBP; systolic blood pressure, SD; standard deviation, SVR; systemic vascular resistance, TAPSE; tricuspid annular plane systolic excursion.

As was expected, the patients with lower LVEF and compromised right ventricular function showed worse outcome.

For the assessment of the adjusted associations between composite of all-cause death/hospitalization and other predictors which had been detected in bivariate analysis, a logistic regression model with backward elimination method was applied. The predictors applied in this model included; age, NYHA class, LVEF, systolic blood pressure, RA pressure, mean PAP, PCWP and CI.

In multivariate logistic regression model, lower age (beta = -0.058, *P* = 0.02, odds ratio = 0.944, 95% CI = 0.897-0.993) as well as NYHA class (beta=1.83, *P* = 0.01, odds ratio = 6.2, 95% CI = 1.6-25) were shown to be independently associated with composite of all-cause death/hospitalization.

## Discussion

This study showed that PWV (as a surrogate for arterial stiffness) may not be a useful prognostic factor in patients with advanced HF who are on guideline directed medical therapy.

The prognostic significance of arterial stiffness has been investigated in many cardiovascular disorders including HF.^1,2,4-7,10-14, 20-25^ Bonapace et al^13^ demonstrated that arterial stiffness is a good predictor of peak Vo2 in patients with dilated cardiomyopathy. Sakuragi et al^15^ and Kaji et al^16^ found a significant relationship between pro-BNP level and aortic stiffness. Demir et al^3^ assessed 98 ischemic cardiomyopathy patients with LVEF ≤35% and NYHA functional class III-IV and revealed a cut off value of 11.06 m/s for PWV as a prognostic factor and predictor of mortality in these patients. Regnault et al^14^ investigated 306 patients of EPHESUS trial. They showed that PP and decreased PWV contribute to cardiovascular outcome. In their study, the mean of PWV was more than 11 m/s.

In the present study we could not find any relationship between PWV and known HF prognostic factors including LVEF, NYHA class and hemodynamic measures. The mean PWV in our study was markedly lower than those observed in similar studies and only 4 patients showed the cut point of 11 mentioned in Demir et al^3^ study. The lower values of PWV in our study population may be an explanation for the lack of relationship between PWV and prognostic factors including death and rehospitalization. The PWV more than 11 m/s has been correlated with prognosis in similar studies and the mean PWV was 6.8 m/s in our study.

Although, it has been shown, PWV is increased in patients with HF whether LV function is impaired or preserved, there was no study reporting the fact that arterial stiffness was investigated in advanced HF. On the other hand, it has been shown that all known cardiovascular pharmacological approaches are capable of decreasing arterial stiffness. The influence of some drugs is uniform which means the drug group definitely improves arterial stiffness and some drugs have prevailing effect that means the drug group improves arterial stiffness in the majority of studies. In this regard, angiotensin converting enzyme inhibitors (ACEIs)/angiotensin receptor blockers (ARBs), calcium channel blockers and spironolactone have uniform effect on arterial stiffness and beta-blockers and statins have prevailing effect.^5,7,26,27^

REASON (preterax in regression of arterial stiffness in a controlled double blind) study was the first study in which the long term influence of ACEIs on arterial stiffness was evaluated. They showed the combination of perindopril/indapamide was efficacious in reducing systolic blood pressure as well as PWV.^28^ Mahmud et al^29^ and Shahin et al^7^ showed ACEIs significantly decrease PWV and arterial stiffness and this effect is independent of their decreasing blood pressure effect. These studies suggest that the effect of these drugs in decreasing mortality in HF patients is through decreasing arterial stiffness.

Beta-blockers have been shown to affect central pulsatile hemodynamics and arterial stiffness by reducing heart rate. The heart rate reduction influences the viscoelastic properties of the arterial wall.^27^

The spironolactone proved to reduce PWV and arterial stiffness when adjusted for blood pressure, compared to bendrofluazide. The beneficial effect of spironolactone in this regard was also observed in patients with non-ischemic dilated cardiomyopathy.^29,30^

All of our study population was treated with a combination of ACEIs or ARBs and beta blockers as well as spironolactone for a long time which could be an explanation for lower PWV in them. The favorable effect of guideline directed medical therapies with ACEIs/ARB, beta blocker and spironolactone on arterial stiffness may modulate the prognostic value of PWV.

Although the drug history has not been mentioned in most of the similar studies including the study done by Demir et al,^3^ the influence of pharmacological therapies on arterial stiffness and different study population can be an explanation for the different findings of our study. It seems that the arterial stiffness has exerted its maximal effects on the heart in advanced HF and at this stage may not have prognostic significance any more.

### Study limitations

The selection of advanced HF patients as study population is the strength of this study, however, there are some limitations. The main limitation of this study was relatively small sample size, particularly in that group with higher NYHA class. The second limitation was relatively short follow up duration and at last in this study we did not assess the Pro-BNP level as a prognostic factor.

## Conclusion

In conclusion, although PWV is a good surrogate for arterial stiffness, it may not be a good prognostic factor in patients with advanced HF. The PWV is not correlated with hemodynamic findings and do not have any added value over previous well known prognostic factors such as CO and NYHA class in patients with advanced HF.

## Competing interests

The authors declare that they have no competing interests.

## Ethical Approval

The study was approved by the ethic and research committee of Rajaie cardiovascular medical and research center and informed consent was obtained from all the participants.

## Acknowledgments

We would like to thank Raymand Rad Company for their kind support in this study and in particular Mr. Karimi. We would also like to thank Dr. Nick Austin for language editing of our paper.
